# The Evolving Landscape of Leptomeningeal Cancer from Solid Tumors: A Systematic Review of Clinical Trials

**DOI:** 10.3390/cancers15030685

**Published:** 2023-01-22

**Authors:** Lina Marenco-Hillembrand, Michael A. Bamimore, Julio Rosado-Philippi, Blake Perdikis, David N. Abarbanel, Alfredo Quinones-Hinojosa, Kaisorn L. Chaichana, Wendy J. Sherman

**Affiliations:** 1 Department of Neurological Surgery, Mayo Clinic, Jacksonville, FL 32224, USA; 2 Department of Neurological Surgery, Cooper University Hospital, Camden, NJ 08103, USA; 3 College of Arts and Sciences, Duke University, Durham, NC 27708, USA; 4 Department of Neurology, Mayo Clinic, Jacksonville, FL 32224, USA

**Keywords:** leptomeningeal cancer, carcinomatous meningitis, neoplastic meningitis, clinical trial

## Abstract

**Simple Summary:**

Leptomeningeal carcinomatosis is a devastating complication of solid malignancies and can occur concurrently in patients with brain metastasis. Despite progress in the treatment of brain metastasis, the survival of patients with leptomeningeal cancer remains stagnant. In the present work, we aimed to conduct a systematic review to evaluate outcome measures, complications, adverse effects, and limitations of therapies explored, with the objective to critically evaluate previous treatments as well as discuss the landscape of ongoing clinical trials for leptomeningeal carciomatosis.

**Abstract:**

Leptomeningeal carcinomatosis (LMC) is a fatal but uncommon complication occurring in 5–15% of patients with stage IV cancer. Current treatment options are ineffective at managing leptomeningeal spread, with a median overall survival (mOS) of 2–6 months. We aimed to conduct a systematic review of the literature to identify past and future therapies for LMC from solid tumors. Forty-three clinical trials (CTs) published between 1982–2022 were identified. Of these, 35 (81.4%) were non-randomized CTs and 8 (18.6%) were randomized CTs. The majority consisted of phase I (16.3%) and phase II CTs (65.1%). Trials enrolled patients with LMC from various primary histology (n = 23, 57.5%), with one CT evaluating LCM from melanoma (2.4%). A total of 21 trials evaluated a single modality treatment. Among CTs, 23.7% closed due to low accrual. Intraventricular (ITV)/intrathecal (IT) drug delivery was the most common route of administration (n = 22, 51.2%) vs. systemic drug delivery (n = 13, 30.3%). Two clinical trials evaluated the use of craniospinal irradiation for LMC with favorable results. LMC continues to carry a dismal prognosis, and over the years, increments in survival have remained stagnant. A paradigm shift towards targeted systemic therapy with continued standardization of efficacy endpoints will help to shed light on promising treatments.

## 1. Introduction

Leptomeningeal carcinomatosis (LMC) occurs due to the dissemination of malignant cancer cells into the leptomeninges (pia and arachnoid) [1,2]. As patients with oncological diseases live longer, the incidence of LMC is becoming higher, with over 5–15% of patients being diagnosed in the United States annually [1,3]. Additionally, innovations in neuroradiologic imaging have contributed to the increasing rate of diagnosis of LMC [3,4]. Despite advances in cancer therapeutics, the prognosis of patients with LMC is currently dismal, with a median overall survival (mOS) of 2–4 months with current therapies and 1–1.5 months if left untreated [3]. Because of the high percentage of mortality seen in LMC, achieving optimal palliative care is the therapeutic objective of multidisciplinary teams, with prolonging survival as a secondary objective in these patients [1,2,5]. However, novel therapies have emerged with the objective to improve the survival and delay the neurological progression of patients with LMC. We aim to systematically review past and ongoing clinical trials of leptomeningeal carcinomatosis from solid tumors as well as discuss the effect of available therapeutic modalities on outcome measures of LMC, including the promise and limitations of actively enrolling clinical trials.

## 2. Materials and Methods

### 2.1. Search Strategy

The systematic review of the literature was registered in the International Platform of Registered Systematic Reviews and Meta-Analysis Protocols (INPLASY), INPLASY2022120112, and conducted adhering to the Preferred Reporting Items for Systematic Reviews and Meta-Analysis (PRISMA) guidelines and recommendations [6]. PubMed, Scopus, and Ovid (Embase) were queried without restrictions of publication language. The search terms “Leptomeningeal Carcinomatosis” OR “Carcinomatous Meningitis” OR “Leptomeningeal Metastasis” OR “Neoplastic Meningitis” AND “Clinical Trial” were used to search these databases from inception to December 2022. Additional publications were identified from the reference list of selected papers.

The Population Intervention Comparator Outcome Study (PICOS)-designed framework was used to structure the research question for the review [6]. Specifically, the research question was: Among adult patients with LMC from solid tumors (population) treated with chemotherapy, targeted therapy, or immunotherapy (intervention and comparator), what are the differences in overall survival (OS) and progression-free survival (PFS) and treatment response based on clinical trial outcomes?

### 2.2. Eligibility Criteria

Included articles reported (1) human subjects ≥ 18 years, (2) diagnosis of LMC from solid tumors confirmed by imaging or cerebrospinal fluid (CSF) cytology and clinical or neurological symptoms, and (3) clinical trials, (4) with either PFS or OS outcomes listed. Book chapters, case reports, review articles, observational studies, editorials, and publications of LMC from hematological tumors and studies consisting solely of pediatric patients were excluded from the analysis.

### 2.3. Data Extraction

The studies retrieved were individually screened by two reviewers (L.M.H. and M.A.B.). for eligibility. Discrepancies between studies included were resolved by discussion or adjudicated by a third observer. Data collection was performed independently by four reviewers (L.M.H., M.A.B., J.R.P., B.P.). Adhering with PRISMA guidelines, data were extracted directly from the article text, tables, and figures. The following variables were collected: study design, primary cancer histology, clinical trial phase, randomization, number of patients, gender, median age, diagnostic criteria for LMC, median Karnofsky performance score (KPS), presence of advanced systemic disease, concomitant brain metastasis, previous radiotherapy or chemotherapy, concomitant systemic chemotherapy, concomitant radiotherapy, treatment modality (systemic therapy or intrathecal therapy), drug class, adverse events, and risk of bias. The main outcome measures were OS from LMC defined as the time for LMC diagnosis to death due to any cause or at last follow-up and PFS defined as the time from diagnosis of LMC to the first documentation of disease progression or death. Identifiable favorable prognostic factors according to primary tumor histology were also recorded. Regarding recorded adverse events, Grade 3 or higher toxicities were defined according to Common Terminology Criteria for Adverse Events 4.0 [7]. Grade 3 complications were defined as disabling, severe or medically significant complications that require hospital admission or prolonged hospitalization. Grade 4 complications are defined as potentially life-threatening complications and require urgent medical intervention.

## 3. Results

### 3.1. Literature Search

**Figure 1:**
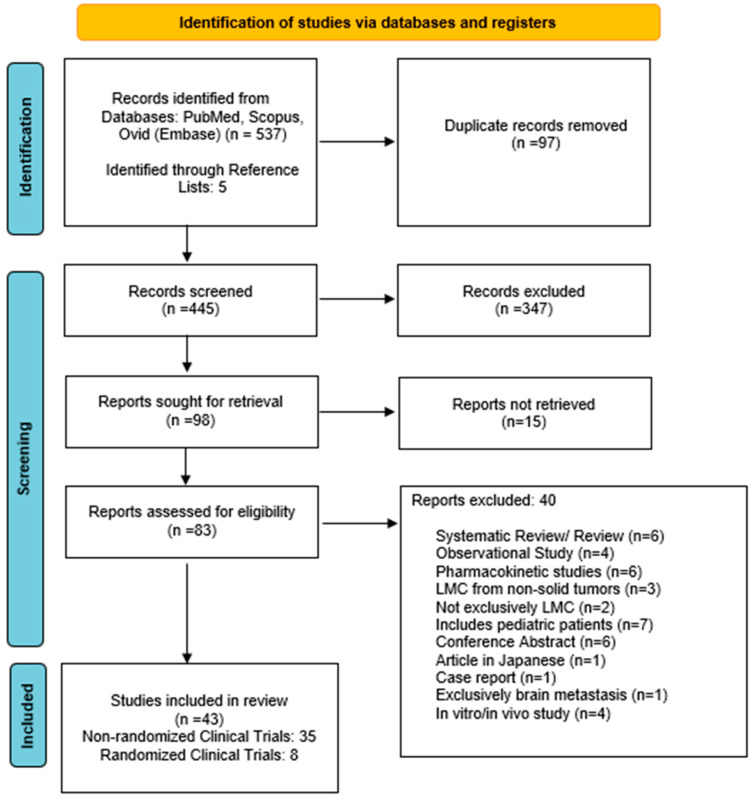
Preferred Reporting Items for Systematic Reviews and Meta-Analysis (PRISMA) search strategy.

The search strategy yielded 537 articles. Following the removal of non-relevant publications, a total of 83 reports were assessed for eligibility (Figure 1). Forty-three clinical trials published between 1982–2022 were included in the qualitative synthesis.

### 3.2. Clinical Trials

**Figure 2:**
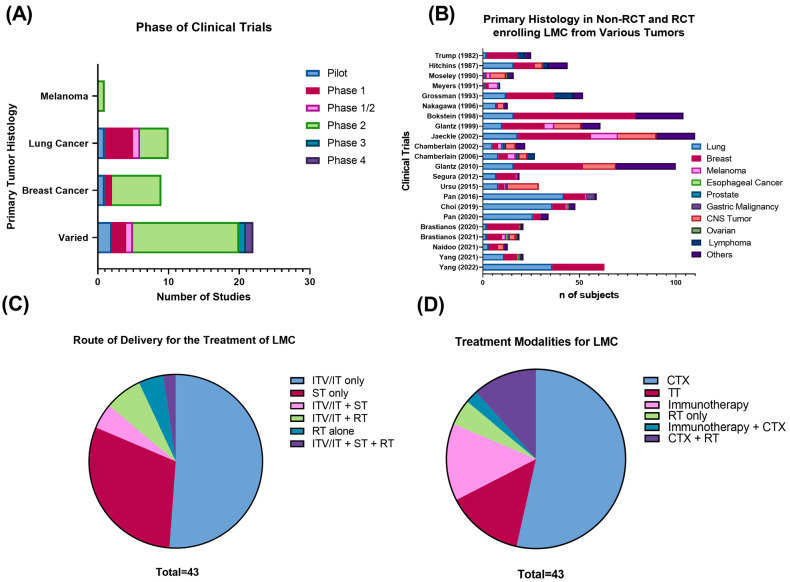
(**A**) Phase of clinical trials of LMC from solid tumors included in the systematic review. (**B**) Primary tumor histology in non-RCT and RCT enrolling LMC from various types of tumors. (**C**) Route of administration and (**D**) treatment modality for published clinical trials of LMC from solid tumors. Abbreviations: CTX: cytotoxic chemotherapy; ITV: intraventricular; IT: intrathecal; LMC: leptomeningeal cancer; RT: radiotherapy; RCT: randomized controlled trial; TT: targeted therapy.

Forty-three clinical trials published between 1982–2022 were included in the qualitative synthesis. The majority consisted of non-randomized (n = 35, 81.4%), phase I (n = 7, 16.3%), or phase II (n = 28, 65.1%) clinical trials (Figure 2A). Lung (25%) and breast cancer (22.5%) were the most frequent primary histology in trials enrolling LMC from a specific primary histology. There was only a single, non-randomized phase II clinical trial focused on evaluating multimodality treatment for LMC from melanoma (n = 1, 2.4%) [8]. A total of 23 trials (57.5%) enrolled patients with LMC from diverse primary tumors, where breast (35.4%), lung (31.4%), and other (14%) were listed as the most common types of histology (Figure 2B).

The most frequent route of administration of therapy was the intraventricular (ITV) and intrathecal (IT) route (n = 22, 51.2%) (Figure 2C). Cytotoxic chemotherapy was the most common therapeutic modality (53.5%), followed by targeted therapy (14%) and immunotherapy (14%) (Figure 2D).

### 3.3. Cytotoxic Chemotherapy

#### 3.3.1. Non-Randomized Clinical Trials

The most extensively studied therapeutic modality for LMC is cytotoxic chemotherapy, either delivered by intraventricular/intrathecal (ITV/IT) administration, systemically, or in combined systemic/intra-CSF treatment regimens (Table 1). Studies evaluating chemotherapeutic agents comprise over half of the published clinical trials for LMC [8,9,10,11,12,13,14,15,16,17,18,19,20,21,22,23,24,25,26,27,28,29,30,31,32], most of which evaluate the efficacy of ITV/IT single-agent chemotherapy (50%). In these trials, the two drugs that have been most thoroughly studied are ITV/IT methotrexate (MTX) (12.5%) and cytarabine (Ara-C)/liposomal cytarabine (DepoCyt) (18.8%). These two chemotherapeutics have shown a marginal success rate in prolonging OS, with pooled mOS of 5.9 and 5.26 months for clinical trials evaluating the use of ITV/IT MTX or Ara-C/DepoCyt, respectively [11,12,13,14,15].

Other chemotherapeutic drugs tested as alternatives for treating LMC are intra-CSF pemetrexed and etoposide [16,17,18,19]. Like MTX, pemetrexed is a folate antimetabolite and acts by inhibiting three key enzymes that participate in folate metabolism. This prevents folate-dependent purine and pyrimidine biosynthesis, exerting its antineoplastic effects by inhibiting DNA and RNA formation in cancer cells [43]. The use of pemetrexed has been mainly evaluated in tyrosine kinase inhibitor (TKI)-failed NSCLC LMC [16,17,18]. An initial pilot study tested the feasibility, safety, and maximum tolerated dose of IT pemetrexed in 13 patients with EGFR mutant NSCLC refractory LMC [22]. For the 11 patients who completed induction chemotherapy, the median PFS (mPFS) was of 2.5 months (CI 0.3–12.5), and mOS was 3.8 months (CI 0.3–14). Severe side effects included radiculitis, hepatotoxicity, and myelosuppression, which were diminished by adding supplemental folinic acid and B12 to pemetrexed therapy [22]. A follow-up phase 1/2 trial examined the safety and efficacy of pemetrexed in a larger cohort of 30 participants with refractory NSCLC LMC. This study reported a promising survival of 9 months (95% CI: 6.6–11.4 months) and a clinical response rate of 84.5% (22/26), with 18 patients reporting higher KPS scores after treatment [17]. Interestingly, positive outcomes were independent of a negative CSF conversion, which only occurred in two patients. These two trials suggest the IT pemetrexed has an acceptable safety profile and may be a reasonable choice for treating TKI-failed NSCLC LMC [17]. Moreover, the use of IT pemetrexed with concomitant involved field radiotherapy (IFRT) has shown adequate safety and efficacy in patients with LMC from solid tumors in a recent phase 1/2 study [30]. Topoisomerase inhibitors, mainly the use of intra-CSF etoposide, have been evaluated for treating leptomeningeal metastasis from diverse primary histology. A phase II trial including 27 participants with LMC from different primary tumors reported a 26% complete or partial response rate with IT topotecan. Although IT topotecan failed to increase OS over existing therapy (mOS 2.5 months), treatment responders presented a longer mPFS of 4.6 months with an acceptable safety profile [35]. The efficacy of other topoisomerase inhibitors such as irinotecan has been evaluated concomitantly with temozolomide (TMZ) in a phase II study including patients with progressing brain metastasis with or without LMC from breast cancer [10]. This trial included a subgroup of eight patients with LMC which presented a median OS of 3 months following treatment. Overall, the most common serious adverse effects experienced with irinotecan and TMZ were neutropenia, nausea, and fatigue [10]. As a standalone therapy for LMC, temozolomide was explored in a phase II non-randomized study for patients with LMC from breast, NSCLC, and melanoma [34]. A total of 19 patients were enrolled in the study, of which 16 progressed (86%). Treatment with TMZ did not confer a significant survival advantage as subjects had a median OS of 1.41 months. Although TMZ was well tolerated, the clinical response was modest as only two patients responded, one with a partial response and the second patient with stable disease.

Recent chemotherapy trials for LMC focus on optimizing methods of drug delivery into the CNS that minimize the off-target effects from chemotherapy. An example is paclitaxel trevatide (ANG10005), a brain-penetrating paclitaxel/angiopep-2 drug conjugate tested in a recent phase II trial [9]. Its intravenous administration to breast cancer patients with LMC was linked to a mPFS of 3.4 months and a mOS of 8.0 months, which exceeded values previously reported in series using IT MTX or systemic chemotherapy [9]. Moreover, treatment with ANG10005 resulted in a promising 3-month PFS rate of 83% and a 6-month survival rate of 63% [9]. Although the primary endpoint of intracranial objective response rate (iORR) was not met, due to the favorable treatment effects experienced by the LMC group, a randomized phase III trial comparing ANG1005 to the best treatment of choice is underway to evaluate its efficacy in LMC (NCT03613181). However, despite the increased brain penetration of this drug conjugate, most subjects experienced severe systemic side effects from the use of ANG10005, most commonly myelosuppression, which led to dose reduction in a third of the participants [9].

#### 3.3.2. Randomized Clinical Trials (RCTs)

Of the eight published RCTs, the majority evaluated the use of cytotoxic chemotherapy in LMC from solid tumors (Table 2). Most compared the safety and efficacy between ITV MTX and DepoCyt [44,45,46]. Although initial trials showed similar safety and efficacy profiles between ITV MTX and DepoCyt, without differences in treatment response (*p* = 0.76) or mOS (*p* = 0.15), patients treated with ITV DepoCyt experienced delayed neurological progression (*p* = 0.007) and longer meningitis-specific survival (*p* = 0.074) [44]. Similar results were observed in a subsequent phase 2 trial, where patients in the ITV DepoCyt arm had significantly longer PFS values (71 days) (*p* = 0.004) [45]. Due to the increased mPFS, similar mOS, and its less demanding dose schedule, these results indicated a possible advantage of using ITV liposomal cytarabine over MTX in treating LMC.

A single RCT evaluated the efficacy of administering single-agent vs. combination IT chemotherapy in 44 patients with LMC from various types of primary tumors [47]. The patients were randomized to receive IT MTX alone or IT MTX combined with Ara-C [47]. Overall, both groups had similar mOS, negative cytologic conversion rates, and clinical response [47]. In regard to toxicity, the incidence of adverse effects was greater in the combined MTX/Ara-C arm without statistical significance. Only one RCT evaluated ITV MTX to the alkylating agent thiotepa delivered via an Ommaya reservoir, without any significant differences in survival or progression [48]. Overall, both groups had similar mOS, negative cytologic conversion rates, and neurological symptom improvement. However, patients on MTX experienced significantly more neurologic (*p* < 0.0008) and skin/mucous membrane complications (*p* = 0.042) as well as increased emesis compared to thiotepa (*p* = 0.08) [48].

**Table 2:** Randomized clinical trials for leptomeningeal carcinomatosis from solid non-hematological tumors.

Author (Year)	Primary Tumor	Phase	Treatment	(n)	Age (Years)	Median KPS/ECOG	BM (n)	mPFS (Months)	mOS (Months)	Response Criteria	Response Rate
Le Rhun (2019) [49]	Breast	2	Systemic treatment alone	37	47.5	80	15	2	4	Neurological clinical evaluation, MRI, CSF negative conversion	Clinical improvement (1), MRI response (3), complete CSF response (5)
			Systemic treatment + IT liposomal cytarabine	36	50.9	80	8	2.4	7.3		Clinical improvement (6), MRI response (7), complete CSF response (10)
Boogerd (2004) [50]	Breast	2	ITV MTX + systemic chemotherapy and IFRT	17	NA	64	2	5.2	4.2	Neurological clinical evaluation	Improvement (7), stable (3), no response (7)
			Systemic chemotherapy and IFRT	18	NA	71	1	5.5	6.9		Improvement (7), stable (5), no response (6)
Yang (2022) [51]	Varied	2	pCSI	42	57	80	28	7.5	9.9	Neurological clinical evaluation, imaging (stable)	RR: 30/42 (71.4%)
			IFRT	21	61	80	15	2.3	6		RR: 5/21 (23.8%)
Cole (2003) [45]	Varied	2	ITV MTX	30	49	NA	NA	1	2.56	Quality-adjusted survival without symptoms or toxicity (Q-Twist) in days	70 days
			ITV DepoCyt	31	49	NA	NA	1.9	3.45		131 days
Glantz (2010) [46]	Varied	4	ITV MTX	48	NA	NA	20	1.23	NA	Doubling of PFS between groups	-
			ITV DepoCyt	52	NA	NA	23	1.15	NA		-
Glantz (1999) [44]	Varied	2	IT MTX	30	49	70	NA	0.986	2.6	Clinical response, negative conversion of CSF cytology	RR: 20%
			IT DepoCyt	31	49	60	NA	1.9	3.5		RR: 26%
Grossman (1993) [48]	Varied	2	ITV MTX	28	NA	2	NA	NA	3.6	Clinical response, negative conversion of CSF cytology, neuroimaging (CT and myelography)	Complete RR: 21%
			ITV thiotepa	24	NA	2	NA	NA	3.24		Complete RR: 4%
Hitchins (1987) [47]	Varied	2	IT MTX	22	55	NA	6	NA	2.7	Clinical response, negative conversion of CSF cytology, neuroimaging (CT and myelography),	RR: 61%
			IT MTX + Ara-C	20	55	NA		NA	1.6		RR: 45%

Abbreviations: BM: brain metastasis; ECOG: Eastern Cooperative Oncology Group; IFRT: involved field radiation therapy; IT: intrathecal; ITV: intraventricular; KPS: Karnofsky performance score; mOS: median overall survival; mPFS: median progression-free survival; MTX: methotrexate; NA: not available; pCSI; proton craniospinal irradiation; RT: radiation therapy; ST: systemic therapy.

#### 3.3.3. Impact of the Route and Rate of Intra-CSF Administration in Leptomeningeal Cancer

Initial RCTs showed a marginal efficacy of intra-CSF chemotherapy against LMC. The poor therapeutic response observed in early trials was partly attributed to the different efficacy between the routes used for drug delivery. Currently, the two methods used to administer intra-CSF chemotherapy are ITV/IT drug delivery. The traditional method used to administer chemotherapeutics was IT delivery using lumbar puncture (LP). However, ITV administration confers several advantages over IT drug delivery. ITV drug delivery provides a direct and reliable drug distribution into the subarachnoid space and theoretically would be more efficient at treating LMC. Although previous pharmacokinetic/pharmacodynamic studies have found ITV to be superior, few trials have tested the clinical implications of this hypothesis [46]. A phase IV trial analyzed the efficacy of chemotherapy depending on the route of administration by evaluating the effect on PFS when chemotherapy (MTX or DepoCyt) was delivered by Ommaya reservoir vs. LP [46]. Interestingly, there was no difference in mPFS between lumbar or ITV delivery in the sustained release cytarabine (DepoCyt) arm (*p* = 0.35). However, patients treated with MTX had a longer mPFS if it was administered intraventricularly rather than by lumbar puncture (19 vs. 43 days, *p* = 0.048) [46]. Investigators attributed these findings to the shorter half-life of MTX within the CSF, theorizing that when it was administered by LP it did not allow sufficient time for it to adequately diffuse into the ventricles. Another variable believed to influence the rate of adverse events is the perfusion rate used in ventriculolumbar chemotherapy. A clinical trial set out to determine if a slower ventriculolumbar perfusion rate would decrease the constitutional side effects [31]. Ultimately, their findings favored the use of slower perfusion rates (15 mL/h vs. 20 mL/h) as they significantly reduced moderate to severe confusion (*p* = 0.017), nausea and vomiting (*p* = 0.08), and normalized intracranial pressure in 59% of the participants [31].

#### 3.3.4. ITV vs. Systemic Chemotherapy and the Utility of Combination Chemotherapy

A RCT has compared ITV/IT vs. systemic chemotherapy in LMC from solid tumors. This study tested the efficacy of IT MTX plus systemic chemotherapy and IFRT vs. systemic chemotherapy and IFRT [50]. Although there were no differences between neurological symptom progression or survival between groups (*p* = 0.32), the ITV chemotherapy arm presented higher rates of treatment-associated complications including increased moderate headaches, serious gait disturbances, and moderate cognitive impairment (*p* = 0.0072) [50]. Additionally, the IT chemotherapy group had an increased incidence of specific treatment-related complications, the two most common being chemical meningitis and Ommaya reservoir revision. Overall, the trial concluded that ITV chemotherapy did not confer a therapeutic advantage over systemic therapy and that its use was associated with increased neurotoxicity [50].

The utility of ITV/IT DepoCyt and concomitant systemic chemotherapy vs. systemic chemotherapy alone was evaluated in a large phase III RCT in patients LMC from breast cancer [49]. As evidenced in previous reports, adding ITV therapy did not impact mOS but prolonged leptomeningeal mPFS (*p* = 0.04) [49]. However, despite lengthening leptomeningeal PFS, it failed to confer any benefit to OS or improved quality of life in the experimental arm. Moreover, patients who received ITV DepoCyt had increased rates of systemic infections (39% vs. 25%) and 3 patients presented chemical meningitis. However, the increased rate of systemic infections in the experimental arm was attributed to systemic therapy and not thought to be associated with the use of ITV DepoCyt [49].

### 3.4. Targeted Therapy

#### 3.4.1. NSCLC: EGFR TKI Inhibitors

The first trials to test the use of TKIs in LMC were performed using the first-generation TKIs, erlotinib and gefitinib. Both drugs showed an adequate safety profile but only a modest response in treating LMC, with nearly identical mPFS values of 2.2–2.3 months and an OS survival of 3.5 months (Table 1) [18,24]. A small, multicentric phase I trial evaluated the use of the second-generation TKI afatinib (40 mg/day) in 11 EGFR-mut NSCLC patients with LMC. Overall CNS penetration rates resembled that of erlotinib, and mPFS and mOS values were comparable to first-generation TKIs at 2.0 and 3.8 months, respectively [18,23].

Overall, the most promising results have been reported in trials evaluating the use of the third-generation TKI osimertinib (Table 1) [37,38,39]. The phase I BLOOM trial assessed the use of osimertinib in 41 patients with EGFR-mutated NSCLC LMC who had progressed on prior TKI therapy [20]. All patients received osimertinib (160 mg orally once daily) until disease progression or uncontrollable drug-related toxicity [20]. The overall leptomeningeal response rate, determined by response evaluation criteria in solid tumors (RECIST), was 62%. Of the LMC patients with an abnormal neurologic baseline, 57% improved neurologically after treatment. The mPFS and mOS in this group were 8.6 and 11.0 months, respectively. It was estimated that 24% of adverse events were directly linked to osimertinib, with 22% resulting in drug cessation and 12% in dose decrease. One patient suffered from pneumonitis, which was the only serious adverse event attributed to osimertinib. Following the BLOOM study, a multicentric phase II study evaluating the use of osimertinib (160 mg) administered to patients with EGFRm NSCLC LMC found similar results [19]. Overall, their intracranial response rate was 92.5% with mPFS and mOS survival values of 8 and 13.3 months, with similar adverse effects to those encountered in the BLOOM study but few severe adverse events. However, the study was limited by the heavy co-treatment of 62.5% of patients [19].

Alternatively, the use of standard-dose osimertinib (80 mg) has been evaluated as a standalone therapy or combined with bevacizumab (7.5 mg/kg IV) in two trials [16,21]. An initial pilot study investigated the efficacy of standard-dose osimertinib in 13 patients with refractory LMC from EGFR mutant NSCLC. Its use was generally well tolerated, without any grade 3 adverse events and a clear efficacy, evidenced by a mPFS of 7.2 months after therapy. Despite the positive results, due to the small sample size, further studies are needed to evaluate the clinical efficacy of standard-dose osimertinib for refractory LMC. The efficacy and safety of administering osimertinib with bevacizumab has been assessed in a phase II single-arm trial [16].. As both drugs penetrate the blood–brain barrier (BBB) and are equally efficacious in the CNS, it was presumed that their combined use may increase the efficacy of anticancer therapy in LMC [16]. The joint use of these agents resulted in a partial clinical response in 50% of all patients, with a mOS of 12.6 months and a mPFS of 9.3 months. Leucopenia, anorexia, and fatigue were the most common side effects experienced from therapy. Common adverse effects associated with the use of bevacizumab such as hemoptysis/epistaxis were rare, occurring in 3/14 (21%) subjects and were mild (grade 1–2). The results from these findings support the potential use of joint therapy with osimertinib and bevacizumab, as it may confer a clinical benefit to patients with EGFRm NSCLC LMC. However, a notable limitation to generalizing these findings is the small sample size of this study, which consisted of only 14 participants and the lack of a comparator arm of patients treated with only osimertinib.

#### 3.4.2. Breast Cancer: HER2 Targeted Therapy

A phase I trial tested the use of IT trastuzumab in HER2+ breast cancer patients with LMC. The main goal of this study was to determine the feasibility, safety, and dose-limiting toxicity of this drug (Table 1) [12]. In this trial, IT trastuzumab was administered weekly for four weeks at doses of 30, 60, 100, or 150 mg. Overall, IT trastuzumab was well tolerated as all the adverse events reported were mild and included headaches, nausea, vomiting, cervical discomfort, and peripheral neuropathy. Moreover, there were no events of drug-limiting toxicity with IT trastuzumab use. The results of this trial indicated that IT trastuzumab therapy conferred a benefit in survival compared to historical series, as the mOS was 7.3 months after treatment. Following therapy, most patients had either stable or progressive disease according to CSF cytology. However, CSF cytology did not correlate to clinical or radiological response. Due to its feasibility and tolerability, the investigators concluded that the use of IT trastuzumab is reasonable in HER2+ breast cancer patients with LMC and should be given until disease progression. However, these results must be validated in larger phase II trials that specifically evaluate its efficacy.

### 3.5. Immunotherapy

#### 3.5.1. Immune-Checkpoint Inhibitors Nivolumab, Ipilimumab, and Pembrolizumab

At present, immune-checkpoint inhibitors and immunotherapy represent a promising forefront in the treatment of CNS cancer. Specifically, cytotoxic T-lymphocyte antigen 4 (CTLA1) and anti-programed death 1 (PD1) inhibitors have made significant strides in the treatment of parenchymal brain metastases in recent years. However, their role in LMC was unexplored until the recent publication of three single-arm clinical trials. A phase II clinical trial tested the use of combination treatment with ipilimumab and nivolumab in 18 patients with LMC from mixed primary histology [28]. The primary endpoint of this trial was percentage survival at 3 months, while secondary endpoints evaluated toxicity and intracranial and extracranial progression with immunotherapy response assessment for neuro-oncology (iRANO) and RECIST, respectively. Overall, the cohort achieved their primary endpoint with a 44% survival rate at 3 months, while the mOS was 2.9 months, and intracranial and extracranial time to progression were 1.93 and 1.94 months, respectively. In total, 16/18 patients in the study reported an adverse event due to the drug, most commonly nausea, fever, anorexia, or rash. Of these patients, six reported grade 3 or higher adverse events, and 11% terminated protocol therapy due to intolerable toxicity resulting in hepatitis or colitis. Although the mOS in this trial was greater than values in historical series, distinct limitations that preclude the generalizability of these findings include the small sample size as well the concomitant treatment with steroids in most participants (78%), which may have potentially limited the immune response and hindered clinical activity. The use of the anti-PDL1 inhibitor pembrolizumab was studied in another phase II trial by the same group in patients with LMC from various solid malignancies [29]. The treatment protocol consisted of administering pembrolizumab 200 mg IV monotherapy every 3 weeks until progression or unacceptable toxicity. The primary endpoint of 3-month OS was achieved in 60% of patients with a mOS for the entire cohort of 3.6 months and an intracranial and extracranial time to progression of 2.6 and 3.6 months, respectively. The best intracranial response achieved in half of the patients was stable disease with iRANO criteria. Hyperglycemia, nausea, and vomiting were the most common side effects associated with protocol treatment. Although 3-month OS was higher than in the ipilimumab and nivolumab trial, patients treated with pembrolizumab were less functionally impaired as 95% had ECOG between 0–1. Another limitation of this trial was the high percentage of breast cancer histology in 17/20 (85%) of subjects enrolled. A smaller phase II trial evaluated the use of pembrolizumab in 13 subjects with LMC from solid tumors [27]. Overall, 38% of patients presented a CNS response at 12 weeks, with two patients achieving a durable complete CNS response. The median CNS PFS and mOS of patients with LMC treated with pembrolizumab were 2.9 and of 4.9 months, respectively, resembling the values reported in the previous pembrolizumab trial conducted by Brastianos et al. Because of the favorable CNS response rate and adequate safety profile of pembrolizumab, these results warrant further investigation into immune-checkpoint inhibitors for the treatment of LMC.

#### 3.5.2. Anti-VEGF Immunotherapy

Clinical studies have demonstrated increased levels of vascular endothelial growth factor (VEGF) in the CSF of patients with LMC, which correlate to a poor prognosis. Due to these findings, a pilot trial investigated the efficacy of bevacizumab in conjunction with etoposide and cisplatin in subjects with LMC from breast cancer [13]. Patients were given bevacizumab, etoposide, and cisplatin every 3 weeks for a maximum of six cycles or until intolerable toxicity occurred. A total of 8 participants were enrolled; however, three patients withdrew from the study, and only five patients were sampled to determine CNS response. Of these, 3/5 (60%) patients had CNS-specific response. The mOS and mPFS were both 4.7 months. Additionally, bevacizumab did not increase the delivery of etoposide into the CSF. This trial had significant limitations. Overall, the study was underpowered to determine its primary endpoint of efficacy, which became more evident after three subjects withdrew from treatment. Moreover, CNS response was determined solely by CSF and clinical parameters, with no radiological imaging.

#### 3.5.3. Other Types of Immunotherapies

Other types of immunotherapies have been explored in three additional clinical trials, which include the use of IT-administered 131I radiolabeled monoclonal antibodies, toll-like receptor agonists CpG-28, and intraventricularly administered alpha interferon [46,47,48]. These consisted of one pilot study, one phase I trial, and one phase II trial, which enrolled patients with LMC from various types of primary tumors. Overall, the use of CpG-28 toll receptor agonist and 131I radiolabeled monoclonal antibodies was well tolerated; however, further studies are required to verify the result from the pilot and phase I studies [33,41]. Lastly, the use of ITV-administered alpha interferon was evaluated in a phase II study in nine patients with LMC. The results demonstrated that ITV alpha interferon therapy led to severe neurotoxicity and is contraindicated [40].

### 3.6. Radiotherapy

At present, the use of radiotherapy for LMC is indicated for treating nodular disease and symptomatic cerebral or spinal sites [2]. Until recently, few trials addressed the use of radiotherapy for treating leptomeningeal metastasis and only investigated the use of IFRT as an adjuvant to systemic or intraventricular therapy [52]. As LMC involves the entire neuroaxis, current trials evaluate the safety and benefit of proton craniospinal irradiation (pCSI) for LMC [26,51]. When compared to traditional photon radiotherapy, where the photons exit the body anteriorly and expose the anterior organs and spinal column to radiation, the bulk of energy of photons is concentrated in the last few millimeters of their range, causing less damage to these structures [26]. Thus, an initial phase I dose escalation trial sought to determine the dose-limiting toxicity of patients with LMC from solid tumors treated with hypofractionated pCSI [26]. A total of 24 patients with LMC, mostly from lung and breast primaries, were given pCSI with an average follow-up of 11 months. The dose deemed to be safe in these patients was 30 Gy given in 3 Gy fractions. Of the 20 patients evaluable for toxicity analysis, only two experienced dose-limiting toxicities, which included grade 3 fatigue and grade 4 lymphopenia and thrombocytopenia. Moreover, the group displayed a promising median CNS PFS of 7 months (95% CI: 5–13) and median OS of 8 months (95% CI: 6-not reached). Due to these encouraging results, the investigators conducted a follow-up phase II RCT comparing photon IFRT to pCSI, with CNS PFS as the primary endpoint [51]. Twenty-one patients with NSCLC or breast LMC were randomized to receive either IFRT or pCSI. Overall, a significant benefit was observed in CNS PFS in patients receiving pCSI when compared to IFRT (7.5 vs. 2.5 months, *p*: < 0.001) [51]. The investigators also observed a benefit in mOS with pCSI vs. IFRT of (9.9 vs. 6.0 months, *p*: 0.029) and no differences in the rate of grade 3 or 4 treatment-related adverse side effects. It was concluded that treatment with pCSI prolonged median CNS-PFS and OS when compared to standard-of-care photon IFRT without an increase in high-grade adverse events [51].

### 3.7. Review of Actively Enrolling Clinical Trials

**Figure 3:**
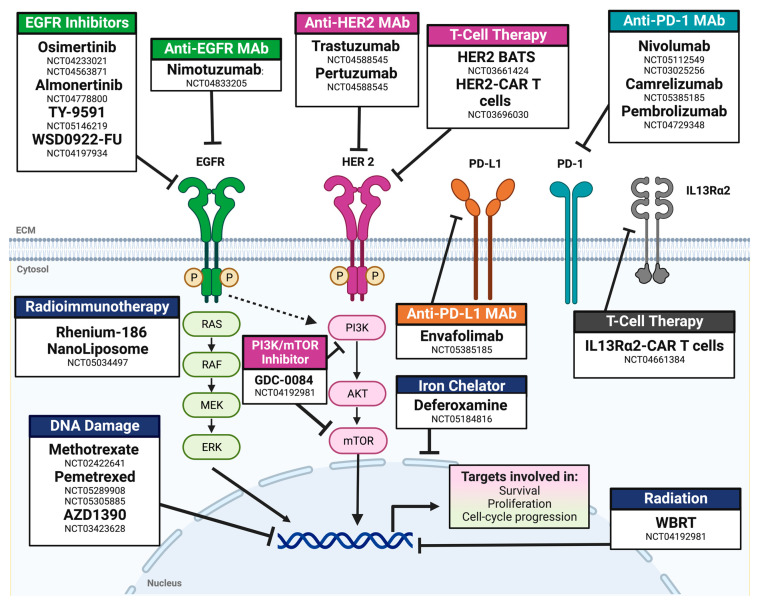
Mechanisms of action for drugs for actively enrolling clinical trials for leptomeningeal carcinomatosis. Abbreviations: Mab: monoclonal antibody; BATS: bi-armed activated T-cells; RT: radiotherapy; WBRT: whole-brain radiotherapy. NCT number: ClinicalTrials.gov identifier.

Twenty-one ongoing clinical trials for LMC were identified. All actively enrolling trials were phase I/II, 45.5% were multicentric, and there was only one RCT evaluating IT pemetrexed and radiotherapy. Of these phase I/II trials, most outcome measures were related to toxicity, safety, or dose finding (63.6%). Of the 21 clinical trials, 90.9% did not have a control arm or were not randomized (95.2%). Most clinical trials included LMC secondary to any primary solid tumor (33.3%), lung (28.6%), and breast (19.0%). A single clinical trial evaluating the role of IV and IT Nivolumab is actively enrolling participants with LMC exclusively from melanoma. In contrast to previously published trials, more than 76.2% of actively enrolling trials evaluated the efficacy of targeted therapy (38.1%), immunotherapy (14.3%), or immune-checkpoint/small-molecule inhibitors (23.8%). The types of therapy being evaluated in current clinical trials are listed in Figure 3.

## 4. Discussion

### Current Limitations for Conducting Clinical Trials in Leptomeningeal Cancer and Future Perspectives

The adequate design and execution of clinical trials for LMC is often challenging [53]. There are important limitations that are inherent to the disease. Although the incidence of LMC is rising due to longer survival rates, it continues to be a rare complication of advanced cancer. Of the clinical trials published on LMC from solid tumors, nine (21.4%) were prematurely closed due to decreased accrual [9,19,20,21,31,34,35,44,53]. Ultimately, the findings from LMC trials with small sample sizes are difficult to interpret and are not generalizable. Although compulsory international clinical trials may seem like a reasonable strategy to increase accrual, studies conducted at an international scale may be subjected to complex or multiple regulatory agency oversight. An alternative includes encouraging interinstitutional collaborations to conduct trials that are sufficiently powered to identify differences in primary or secondary endpoints. Additionally, most clinical trials in LMC from solid tumors were single-arm phase I or II studies with the primary endpoint being analyzed against historical controls. This can potentially lead to incorrect conclusions; thus, an effort towards RCTs should be encouraged. The limited survival of patients with LMC poses a second limitation, as it creates a bias towards enrolling more clinically fit patients. In the present review, only one clinical trial evaluated the treatment of LMC from solid tumors in a cohort of patients with adverse prognostic factors [32].

Another significant limitation to current trials is the standardization of response for LMC, which is often challenging. The RANO proposal for response criteria sought to provide a tool to assess response to therapy through the evaluation of three elements (a standardized neurological exam, CSF cytology or flow cytometry, and radiological evaluation: progressive, stable, or improved) [54]. However, this scale has not been prospectively validated nor uniformly used across clinical trials for LMC after its publication. Some report using modifications of the criteria or alternative tools such as the RECIST, which are inadequate for LMC as neuroimaging features of LMC are often not measurable. With the increasing adoption and availability of quantitative assessment of circulating tumor cells and circulating tumor DNA in the CSF, this has yet to be universally adopted in response criteria.

Additionally, advocating for a universal treatment strategy in all histologic subtypes of LMC may be counterintuitive. Several studies have found that driver mutations in the primary tumor may be different from those within the metastatic site, and response to therapy may vary according to the primary tumor histology, favoring a more personalized and directed approach [55]. Moreover, the most promising survival values in trials for LMC were observed with targeted therapy and systemic treatment. The main limitation of systemic therapy is the blood–brain barrier, although certain agents such as osimertinib have shown great penetration in preclinical studies, which explains its superior efficacy when compared to first- and second-generation TKIs, which have lesser penetration [56]. Other advantages of systemic therapy include targeting other metastatic sites outside of the brain; fewer side effects associated with ITV/IT therapy, such as chemical meningitis; and an overall less invasive method of administration (oral or IV).

An additional strategy that has displayed increased efficacy in clinical trials is pCSI. Although only two trials evaluated pCSI for LMC, the prolonged CNS PFS and OS values observed are encouraging and resulted in early study closure due to the benefit of pCSI over traditional IFRT. Additionally, RT may be a useful adjunct to immunotherapy in patients with LMC, as radiation increases dendritic cell antigen uptake and presentation to prime an adaptative T-cell mediated immune response against the tumor [57]. Moreover, recent data indicate that for brain metastases, RT in combination with immunotherapy significantly prolongs OS vs. immunotherapy alone, regardless of the primary tumor histology [58]. Despite these results, current immunotherapy trials only allowed RT before enrollment or after participation, and the role of RT combined with immunotherapy has yet to be explored in clinical trials of LMC [28,29].

## 5. Conclusions

LMC is a devastating complication and a late manifestation of progressive cancer from solid tumors. Published clinical trials predominantly explore chemotherapy given intrathecally or systemically, with one RCT finding no differences in survival or progression but increased side effects with IT administration. The best treatment response reported was achieved in trials evaluating targeted and systemic therapy, which advocates for a personalized approach of treating LMC. This conclusion is supported by the high percentage of targeted therapy, immunotherapy, and checkpoint inhibitors tested in ongoing clinical trials. Although immunotherapy is at the current forefront in the treatment of brain metastases, survival values did not surpass those reported in chemotherapy trials, although its efficacy may be limited by concurrent steroid use in this population. Additionally, pCSI represents a promising approach for treatment of LMC from different histologic subtypes. Interinstitutional and collaborative efforts must be made to increase accrual as well as standardize efficacy endpoints to find the most effective method to slow the progression and prolong the survival of patients suffering from LMC.

## Figures and Tables

**Table 1:** Non-randomized clinical trials for leptomeningeal carcinomatosis from solid non-hematological tumors.

Author (Year)	Primary Tumor	Phase	Treatment	(n)	Age (Years)	Median KPS/ECOG	BM (n)	mPFS (Months)	mOS (Months)	Response Criteria	Response Rate
Kumthekar (2020) [9]	Breast	2	IV ANG1005	28	47.5	80	28	3.4	8	CNS RECIST v1.1	Intracranial ORR: (15%) stable/improved intracranial disease (77%)
Melisko (2019) [10]	Breast	2	IV irinotecan and TMZ	8	NA	NA	7	NA	3	NA in LMC subset	NA
Mrugala (2019) [11]	Breast	2	IV MTX and IT DepoCyt	3	50	70	2	1.4	8.2	Radiographic criteria (MRI), CSF cytology	NA
Bonneau (2018) [12]	Breast	1	IT trastuzumab	16	57	80	14	NA	7.3	Clinical, radiographic (RECIST v1.1), CSF cytology.	Clinical response: Responsive (3), stable (7), progressive (4), NA (2). Radiological response: Stable (9), progressive (5), NA (2) CSF cytology response: Responsive (2), Stable (6) Progressive (4), NA (4)
Wu (2015) [13]	Breast	Pilot	IV BEEP	8	55	60	7	4.7	4.7	-CSF cytology, clinically stable or improved	3/5 (60%)
Orlando (2002) [14]	Breast	2	Day: 1 IT thiotepa + MTX, IT hydrocortisone, Day 2: IT cytarabine, IT MTX, IT hydrocortisone, and oral folinic acid	13	45	NA	5	NA	2.07	Complete: -CSF cytology + clinically stable Partial: decreased CSF cytology + clinically improved Failure: no decrease in CSF cytology + clinically stable or progression	0
Esteva (2000) [15]	Breast	2	ITV Ara-C	10	49	NA	2	NA	5.7	Complete: -CSF cytology > 4 weeks, clinically responsive Partial: -CSF cytology < 4 weeks, clinically responsive Failure: -CSF cytology, partial remission after 6 weeks, clinical progression after 3 weeks	Response: Complete (2), Partial (4), Treatment failure (3)
Lu (2021) [16]	EGFRmut NSCLC	2	Oral osimertinib (80 mg) + IV bevacizumab	14	61	NA	11	9.3	12.6	RANO LM radiological criteria	Response (7), Stable (6) Progression (1) LM ORR: 50%
Fan (2021) [17]	EGFRmut NSCLC	1/2	IT premetrexed + dexamethasone	30	54	40–60	NA	NA	9	Neurological signs and symptoms and KPS	RR: 87%, stable (1), Progressive (2), not evaluable (3)
Nosaki (2020) [18]	NSCLC	2	Oral erlotinib	21	64	2	NA	2.2	3.4	Negative conversion of CSF cytology	CSF RR: 30%
Park (2020) [19]	EGFRmut NSCLC	2	Oral osimertinib (160 mg)	40	59	1	NA	8	13.3	RECIST v1.1	ICD complete response: 92.5%, ECD complete response: 85%
Yang (2020) [20]	EGFRmut NSCLC	1	Oral osimertinib (160 mg)	41	59	2	29	8.6	11	RECIST v1.1 and RANO criteria	LM ORR: 27%
Nanjo (2018) [21]	EGFRmut NSCLC	Pilot	Oral osimertinib (80 mg)	13	67	2	NA	7.2	Not reached	Clinical response, -conversion of CSF cytology, neuroimaging findings and RECIST v1.1 (extra CNS tumor)	CNS radiological RR: improved (8), stable (3), progressed (1), not evaluable (1). Clinical RR: improved (4), stable (8), worsened (n = 1)
Pan (2019) [22]	Lung	1	IT pemetrexed	13	55	30	NA	2.5	3.8	RANO criteria	Clinical RR: 31% (4/13)
Tamiya (2017) [23]	Lung	1	Oral afatinib	11	66	2	NA	2	3.8	RECIST v1.1	ORR: 27.3%
Jackman (2015) [24]	EGFRmut NSCLC	1	High-dose oral gifetinib	7	51	2	brain	750 mg: 1.9, 1000 mg: 2.5	750 mg: 1.9 1000 mg: 3.7	Clinical neurological improvement, CSF clearance, radiological response (resolution of LM metastasis on MRI)	Clinical improvement (4/7). CSF clearance: 2/7 partial, 1/7 complete
Chamberlain (1998) [25]	NSCLC	2	ITV therapy (MTX: 32, cytarabine: 16, thiotepa: 6)	32	57	90	9	NA	5	Clinical response, -CSF cytology	MTX RR: 17 (43%), second-line Ara-C RR: 8 (50%), third line thiotepa RR: 2 (33%)
Chamberlain (1996) [8]	Melanoma	2	ITV MTX/Ara-C/thiotepa + RT	16	47	80	NA	NA	4	CSF cytology	RR: complete (2), partial (4), progressive (3)
Yang (2021) [26]	Varied	1	pCSI	21	52	70	11	7	8	RANO-LM criteria	6-month CNS RR: 63% 1-year CNS RR: 19%
Naidoo (2021) [27]	Varied	2	IV pembrolizumab	13	57	0	NA	2.9	4.9	Clinical response, -CSF cytology, neuroimaging findings	CNS RR: 38% (5/13) progressive disease: 61.5% (8/13)
Brastianos (2021) [28]	Varied	2	IV ipilimumab and nivolumab	18	54	NA	13	1.94	2.9	3-month OS, iRANO and RECIST v1.1 criteria	iRANO: Complete (1), stable (7),progression (4) not evaluable (6) RECIST: partial (1), stable (3), progression (3), not evaluable (11)
Brastianos (2020) [29]	Varied	2	IV pembrolizumab	20	51.5	NA	NA	2.6	3.6	3-month OS, iRANO and RECIST v1.1 criteria	iRANO: RR stable (11), progressive (5), not evaluable (4) RECIST RR: stable (10), progression (1), not evaluable (9)
Pan (2020) [30]	Varied	1/2	IT pemetrexed + IFRT	34	56	40	6	3.5	5.5	RANO-LM criteria	Clinical RR: 52.9% (18/34) CSF RR: 32% (8/25) Imaging response: 33% (9/25)
Choi (2019) [31]	Varied	2	Slow VLP IT MTX	47	59	70	NA	NA	5.3	ICP normalization	ICP normalized: 13/22 (59%)
Pan (2016) [32]	Varied	2	IT MTX + RT	59	55	40	NA	NA	6.5	Clinical response (KPS, symptoms)	Complete response (14), obvious response (29), partial (8), stable (5), progressive (3)
Ursu (2015) [33]	Varied	1	IT CpG-28	29	56	70	NA	1.75	3.75	Clinical and imaging response	Clinical improvement (4) Radiological response (3)
Segura (2012) [34]	Varied	2	Oral TMZ	19	51	70	NA	0.92	1.4	RECIST, corticosteroid use, clinical response, and CSF cytology	RECIST response: Complete = 0, partial = 2 (11%) stable = 1 (5%), progressive = 16/19 (84%)
Chamberlain (2006) [35]	Varied	2	ITV etoposide	27	55	NA	NA	11% at 6-months	2.5	Clinical response, negative conversion of CSF cytology	Clinically stable and + CSF (+) = 12 (44%). Neurologically stable/improved/CSF (-) = 7/27 (26%) Complete response = 1/27 Partial response = 6/27
Chamberlain (2002) [36]	Varied	2	ITV alpha interferon	22	56	NA	NA	NA	4.14	Clinical response, negative conversion of CSF cytology	Partial response (10), progressive disease (12)
Jaeckle (2002) [37]	Varied	3	ITV/IT DepoCyt	110	50	70	NA	1.8	3.12	Clinical response, negative conversion of CSF cytology	CSF RR: 19/70 (27%, 95% CI: 17–39%). Rate of neurological progression: 69/110 (63%)
Bokstein (1998) [38]	Varied	2	RT + ITV and systemic chemotherapy vs. RT + systemic chemotherapy	104	NA	NA	NA	NA	4	Clinical response, negative conversion of CSF cytology, neuroimaging findings	RT+ ITV + systemic chemo complete RR: 24/28 (86%) RT + Systemic chemo alone: 20/27 (74%) (*p* >0.05)
Nakagawa (1996) [39]	Varied	Pilot	IT VLP	13	54	NA	NA	NA	7	Good: (-) CSF + clinical improvement Moderate: CSF + clinical improvement Minor: CSF or clinical improvement Non-responder: without improvement	Good: 6/13 Moderate: 3/13 Minor: 2/13 None: 2/13
Meyers (1991) [40]	Varied	2	ITV leukocyte α interferon	9	50	NA	NA	NA	4	Negative CSF cytology	CSF RR: 4/9 (44%)
Moseley (1990) [41]	Varied	Pilot	IT 131I radiolabeled mAb	15	NA	NA	0	NA	12	Clinical response, negative conversion of CSF cytology, imaging	Clinical RR: 5/9, CSF RR: 5/9, imaging RR: 5/9
Trump (1982) [42]	Varied	2	ITV thiotepa and MTX + RT	25	50	NA	10	NA	5.29	Clinical response, negative conversion of CSF cytology	Clinical response: complete (4), partial (5), stable (13), progressive (3) CSF: 13/17 complete RR

Abbreviations: BEEP: bevacizumab, etoposide, cisplatin; BM: brain metastasis; CNS: central nervous system; CSF: cerebrospinal fluid; ECD: extracranial disease; ECOG: Eastern Cooperative Oncology Group; ICD: intracranial disease; IFRT: involved field radiation therapy; IT: intrathecal; ITV: intraventricular; IV: intravenous; KPS: Karnofsky performance score; LM: leptomeningeal; LMC: leptomeningeal carcinomatosis; mAb: Monoclonal antibody; mOS: median overall survival; mPFS: median progression-free survival; MRI: magnetic resonance imaging; MTX: methotrexate; NA: not available; NSCLC: non-small cell lung cancer; ORR: objective response rate; pCSI: Proton craniospinal irradiation; RANO: refinement of response assessment in neuro-oncology; iRANO: immunotherapy response assessment in neuro-oncology; RECIST: response evaluation criteria in solid tumors; RR: response rate; RT: radiation therapy; VLP: ventriculolumbar perfusion.
